# Supplementary material for: Oleanolic Acid Acetate Exerts Anti-Inflammatory Activity via IKKα/β Suppression in TLR3-Mediated NF-κB Activation
Source: Molecules. 2019 Nov 5;24(21):4002. doi: 10.3390/molecules24214002 (PMC6866124; doi:10.3390/molecules24214002)
Supplement: Supplementary file 1 [file molecules-24-04002-s001.zip › molecules-619991-supplementary.pdf]

Supplementary Material

Oleanolic acid acetate exerts anti-inflammatory activity via IKKα/β suppression in TLR3-mediated NF-κB activation

Hyung Jin Lim[1,2,a], Hyun-Jae Jang[3,a], Mi Hwa Kim[1,a], Soyoung Lee[1], Seung Woong Lee[1], Seung-Jae Lee[1,*] and Mun-Chual Rho[1,*]

[1] *Immunoregulatory Materials Research Center, Korea Research Institute of Bioscience and Biotechnology, 181 Ipsin-gil, Jeongeup-si, Jeonbuk 56212, Korea*

[2] *Department of Bioactive Materials Science, Chonbuk National University, Jeonju 54896, Korea*

[3] *Natural Medicine Research Center, Korea Research Institute of Bioscience and Biotechnology, 30 Yeongudanji-ro, Cheongju-si, 28116, Korea*

[a] *These authors contributed equally to this study.*

[*]**Corresponding author**:

Seung-Jae Lee, Ph.D and Mun-Chual Rho, Ph.D.

Immunoregulatory Materials Research Center, Korea Research Institute of Bioscience and Biotechnology, 181 Ipsin-gil, Jeongeup-si, Jeonbuk 56212, Korea Tel.: +82-63-570-5267/5230; Fax: +82-63-570-5239.

E-mail addresses: seung99@kribb.re.kr (S.-J. Lee), rho-m@kribb.re.kr (M.-C. Rho)

Contents		Pages
**Experimental**	General procedure; Synthetic Procedure for biotin labeling of OAA	S3
**Figure S1.**	Scheme of OAA labeling with biotin.	S5
**Figure S2.**	HR-ESI-MS spectrum of Biotin-OAA	S6
**Figure S3.**	^{1}H NMR spectrum of Biotin-OAA ($CDCl_3$, 600 MHz)	S7
**Figure S4.**	^{13}C NMR spectrum of Biotin-OAA ($CDCl_3$, 150 MHz)	S8
**Figure S5.**	DEPT-90 spectrum of Biotin-OAA ($CDCl_3$, 150 MHz)	S9
**Figure S6.**	DEPT-135 spectrum of Biotin-OAA ($CDCl_3$, 150 MHz)	S10
**Figure S7.**	COSY spectrum of Biotin-OAA ($CDCl_3$, 600 MHz)	S11
**Figure S8.**	HMQC spectrum of Biotin-OAA ($CDCl_3$, 600 MHz)	S12
**Figure S9.**	HMBC spectrum of Biotin-OAA ($CDCl_3$, 600 MHz)	S13

Experimental

General procedure

^1H, ^{13}C, DEPT-90, DEPT-135 (600, and 150 MHz), and 2D NMR (COSY, HMQC, HMBC) spectroscopic data were recorded on a JEOL JNM-ECA600 spectrometer with $CDCl_3$ as a solvent. The chemical formula of biotinylated oleanolic acid 3-acetate (biotin-OAA) was determined on the basis of HR-ESI-MS data using a Waters SYNAPT G2-Si HDMS spectrometer (Waters, Milford, MA, USA).

Synthetic Procedure for biotin labeling of OAA

We used the following reagents for biotinylation of OAA. (a) $(COCl)_2$, CH_2Cl_2; (b) $H_2N(CH_2)6NHBoc{\cdot}HCl$, $NaHCO_3$, PhH, water; (c) TFA, CH_2Cl_2; and (d) Biotin, EDC, DMAP, DMF. A mixture of OAA (3.0 g, 6.0 mmol) and oxalyl chloride (5 mL) was stirred at room temperature overnight. The solvent was removed *in vacuo*, and the residue was coevaporated with benzene (three times) to produce compound **2**.

Synthesis of compound **3**: A solution of **2** (180 mg, 0.35 mmol) in benzene (10 mL) was added to a solution of 6-[(tert-butoxycarbonyl)amino]hexylamine hydrochloride (380 mg, 1.5 mmol) and $NaHCO_3$ (126 mg, 1.5 mmol) in water (4 mL). The mixture was stirred at room temperature overnight. The organic layer was separated and then diluted with EtOAc (20 mL). The organic extract was washed with water (three times) and then saturated with aqueous NaCl solution (three times), dried over anhydrous $MgSO_4$, and filtered. The solvent was removed from the filtrate in vacuo. The solid was purified by flash column chromatography [hexanes/EtOAc, 1:1, v/v] to produce **3**.

Synthesis of compound **4**: Trifluoroacetic acid (TFA, 1 mL) was added to solution **3** (100 mg, 0.14 mmol) in CH_2Cl_2 (10 mL). The mixture was stirred at room temperature for 1 h. The solvent was removed *in vacuo*, and the residue was coevaporated with benzene (three times) to produce **4**.

Synthesis of compound **5**: 1-Ethyl-3-(3-dimethylaminopropyl)carbodiimide (EDC, 52 mg, 0.3 mmol) was added to a mixture of biotin (82 mg, 0.3 mmol), compound **4** (128 mg, 0.225 mmol) and 4-dimethylaminopyridine (DMAP, 41 mg, 0.3 mmol) in dimethylformamide (DMF). The mixture was stirred at room temperature overnight. The reaction mixture was partitioned between the ethyl acetate and water. The organic extract was washed with water (three times) and then saturated with aqueous

NaCl solution (three times), dried over anhydrous $MgSO_4$, and filtered. The solvent was removed from the filtrate *in vacuo*. The solid was purified by fresh column chromatography [CH_2Cl_2/MeOH, 9:1, v/v] to produce **5**.

Biotin-OAA (**5**): White powder, $C_{48}H_{78}N_4O_5S$; 1H NMR (600 MHz, $CDCl_3$) δ 6.26 (1H, br s, 1′-CONH), 6.23 (1H, br s, 7′-NH), 5.95 (1H, t, *J* = 5.4 Hz, 28-CONH), 5.42 (1H, s, 8′-NH), 5.34 (1H, t, *J* = 3.0 Hz, H-12), 4.49 (1H, m, H-8), 4.46 (1H, m, H-3), 4.29 (1H, dd, *J* = 6.6, 4.8 Hz, H-7′), 3.33 (1H, m, H-1a″), 3.19 (2H, m, H2-6″), 3.13 (1H, m, H-6′), 2.94 (1H, m, H-1b″), 2.88 (1H, dd, *J* = 12.6, 4.8 Hz, H-9a′), 2.72 (1H, d, *J* = 12.6 Hz, H-9b′), 2.48 (1H, dd, *J* = 13.2, 3.6 Hz, H-18), 2.19 (2H, t, *J* = 7.2 Hz, H2-2′), 2.02 (3H, s, OAc-CH_3), 1.96–1.00 (35H, m, overlap), 1.13 (3H, s, CH_3-27), 0.92 (3H, s, CH_3-25), 0.88 (3H, s, CH_3-29), 0.87 (3H, s, CH_3-30), 0.84 (3H, s, CH_3-23), 0.83 (3H, s, CH_3-24), 0.82 (1H, m, H-5, overlap), 0.73 (3H, s, CH_3-26); ^{13}C NMR (150 MHz, $CDCl_3$) δ 178.5, 173.4, 171.2, 163.9, 145.2, 122.8, 81.0, 62.0, 60.4, 55.8, 55.4, 47.7, 47.0, 46.5, 42.5, 42.3, 40.8, 39.6, 39.3, 38.4, 37.9, 37.1, 36.2, 34.4, 33.2, 32.8, 32.5, 31.0, 29.6, 29.5, 28.4, 28.3, 28.2, 27.5, 26.5, 26.4, 26.0, 25.9, 24.0, 23.9, 23.8, 23.7, 21.6, 18.4, 17.2, 16.9, 15.7; HR-ESI-TOFMS: *m/z* 823.5755 $[M+Na]^+$ (calcd for $C_{48}H_{79}N_4O_5S$, 823.5766).

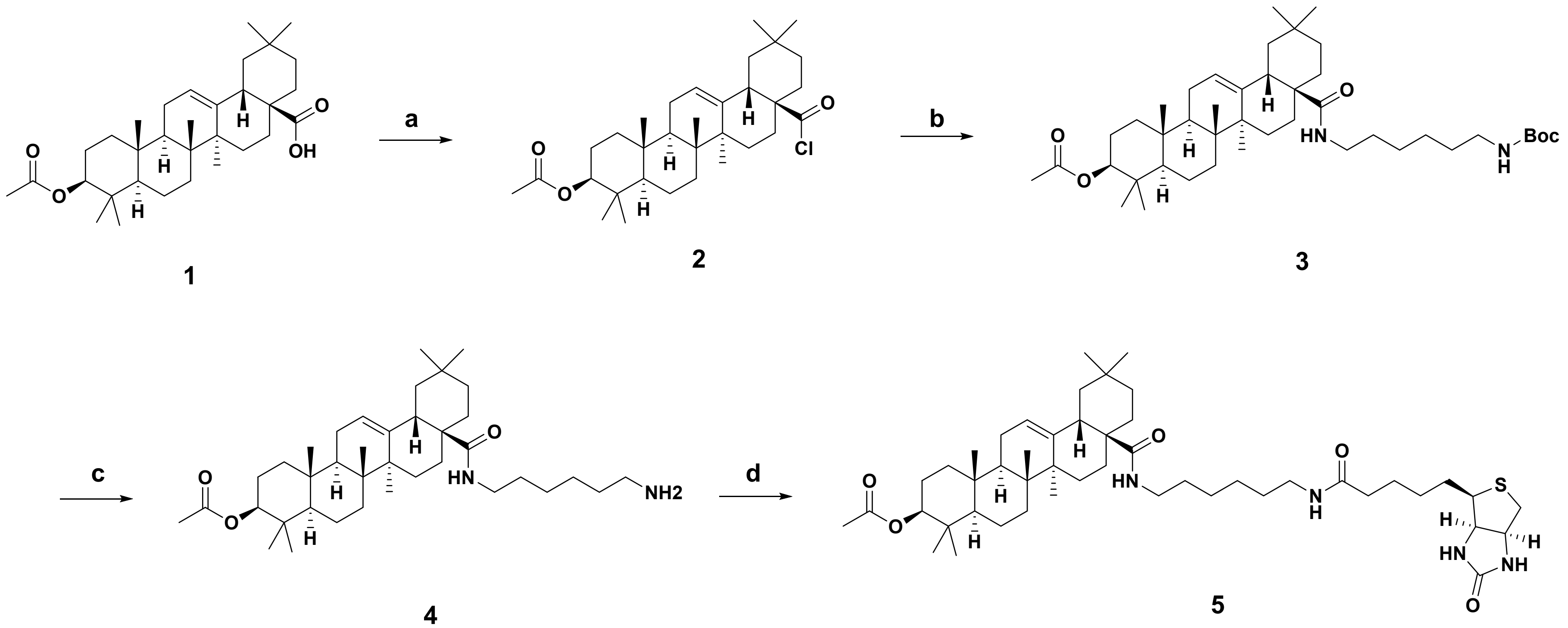

Figure S1. Scheme of OAA labeling with biotin.

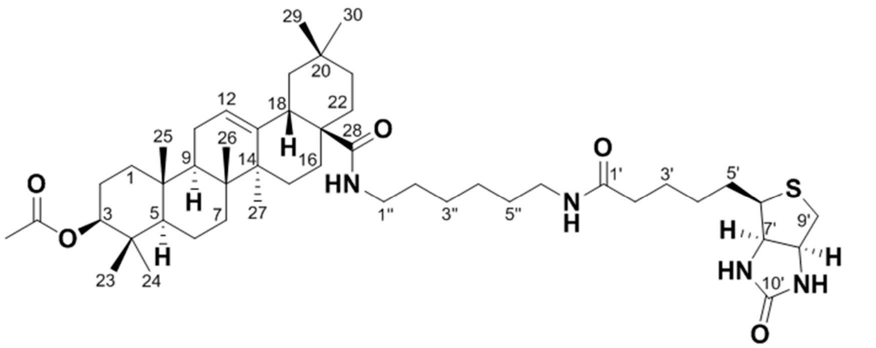

Chemical Formula: $C_{48}H_{79}N_4O_5S^+$, $[M+H]^+$
(Calcd. 823.5766)

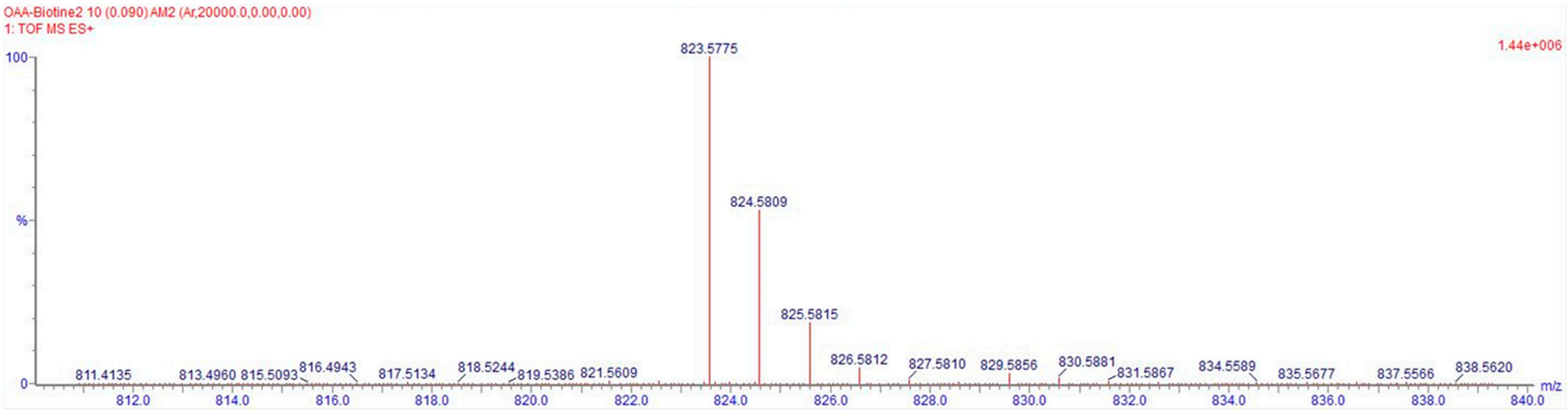

Figure S2. HR-ESI-MS spectrum of Biotin-OAA

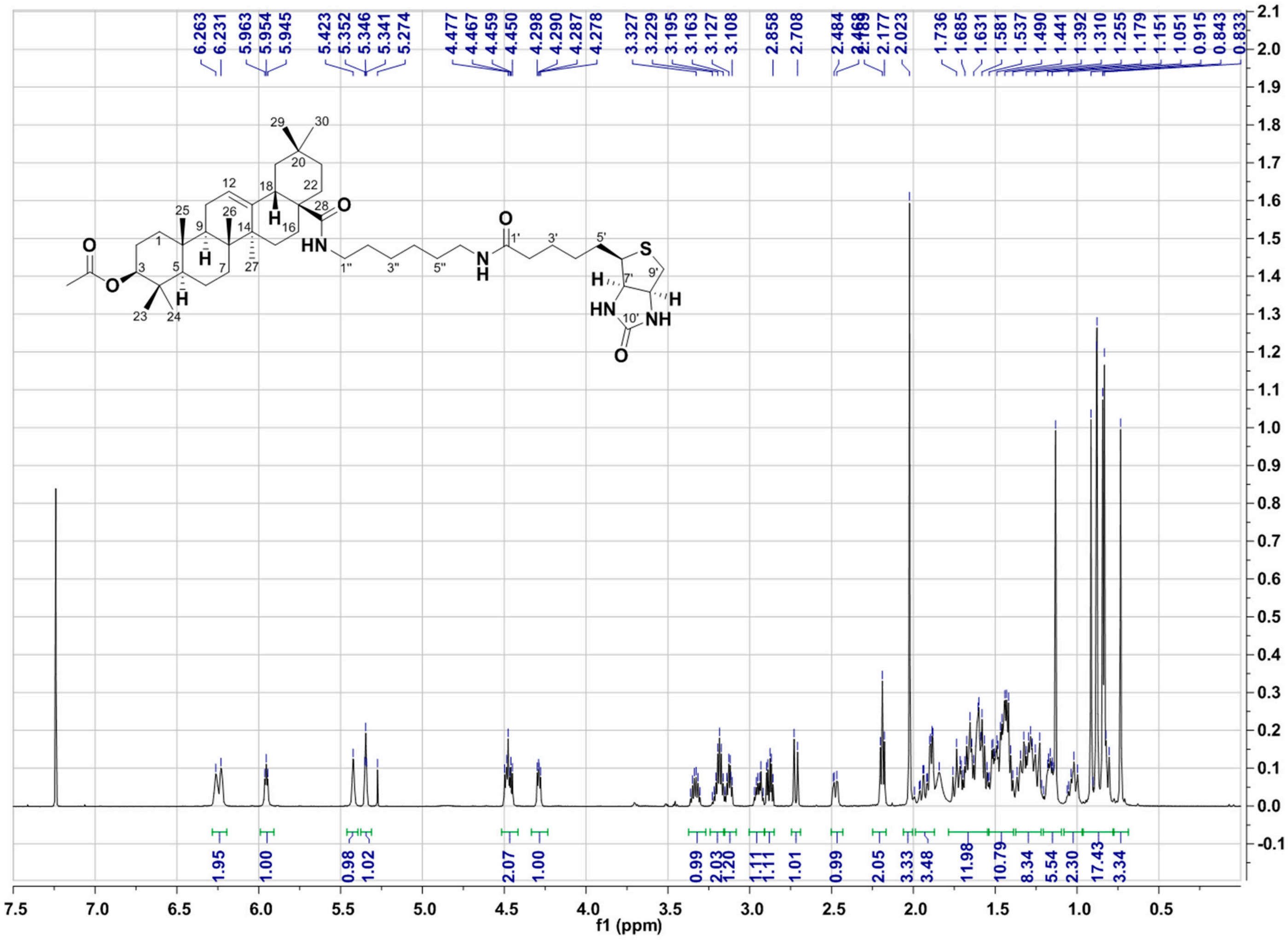

Figure S3. ^{1}H NMR spectrum of Biotin-OAA ($CDCl_3$, 600 MHz)

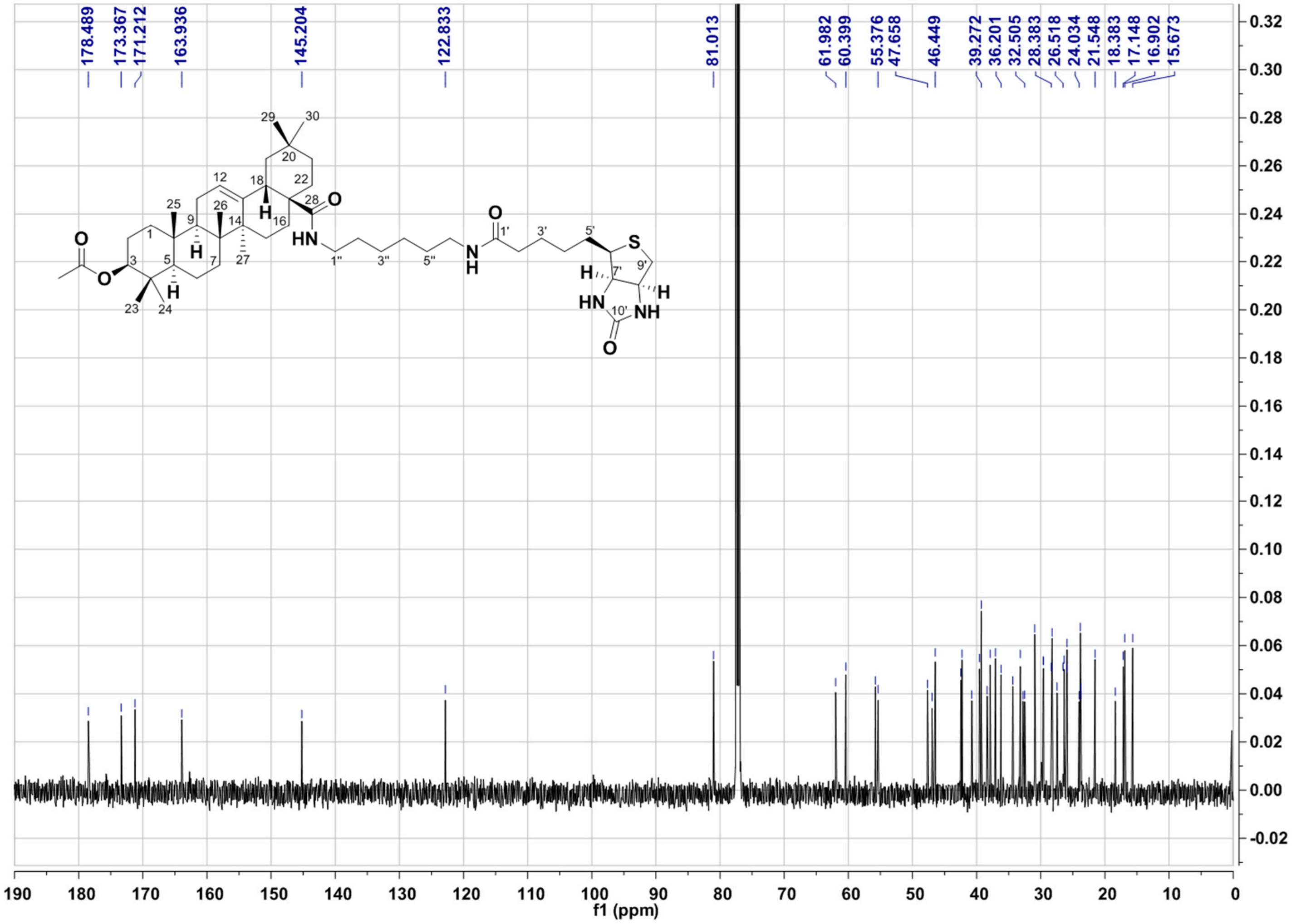

178.489
173.367
171.212
163.936
145.204
122.833
81.013
61.982
60.399
55.376
47.658
46.449
39.272
36.201
32.505
28.383
26.518
24.034
21.548
18.383
17.148
16.902
15.673
f1 (ppm)

Figure S4. ^{13}C NMR spectrum of Biotin-OAA ($CDCl_3$, 150 MHz)

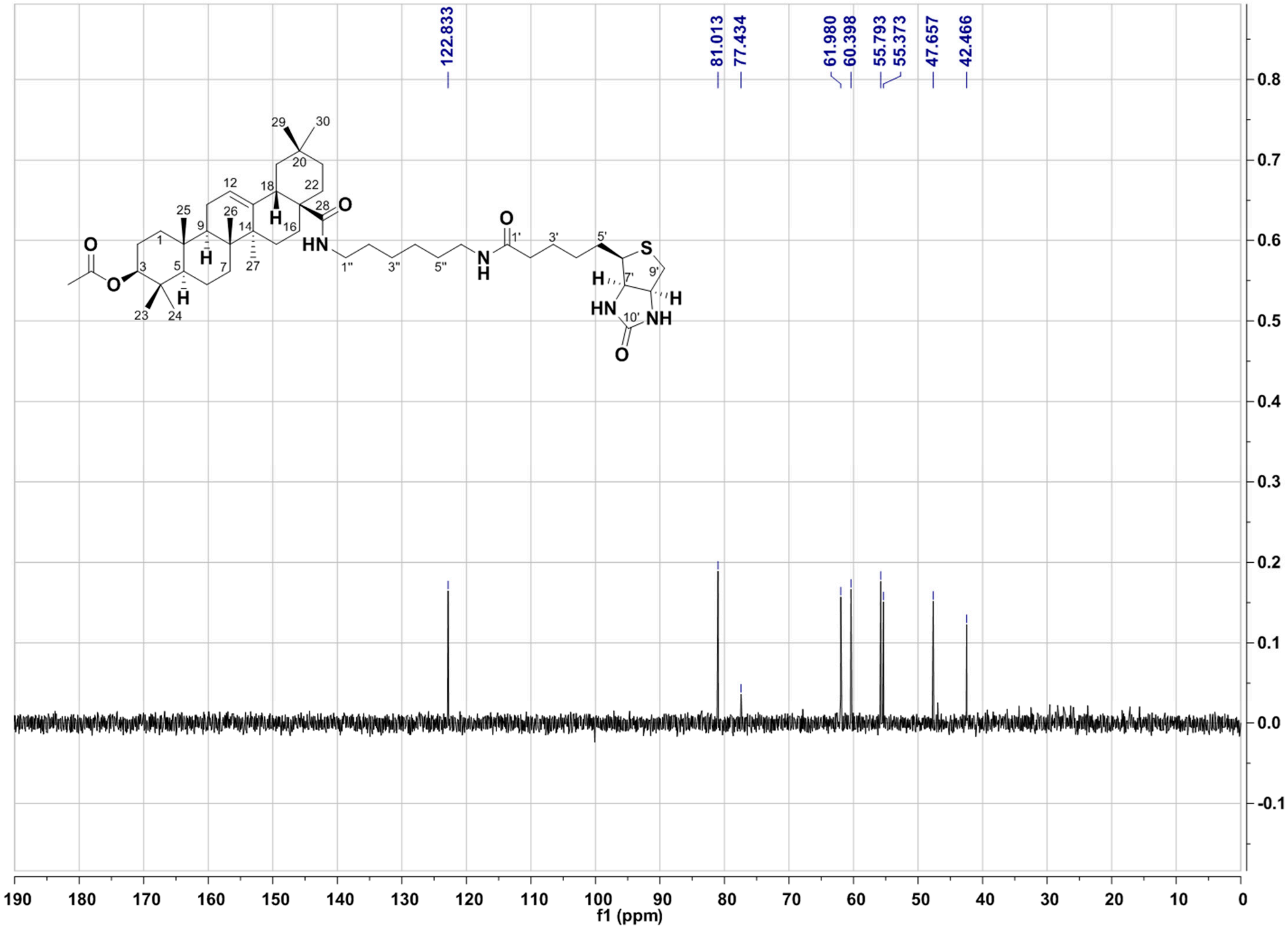
122.833
81.013
77.434
61.980
60.398
55.793
55.373
47.657
42.466
0.8
0.7
0.6
0.5
0.4
0.3
0.2
0.1
0.0
-0.1
190 180 170 160 150 140 130 120 110 100 90 80 70 60 50 40 30 20 10 0
f1 (ppm)

Figure S5. DEPT-90 spectrum of Biotin-OAA ($CDCl_3$, 150 MHz)

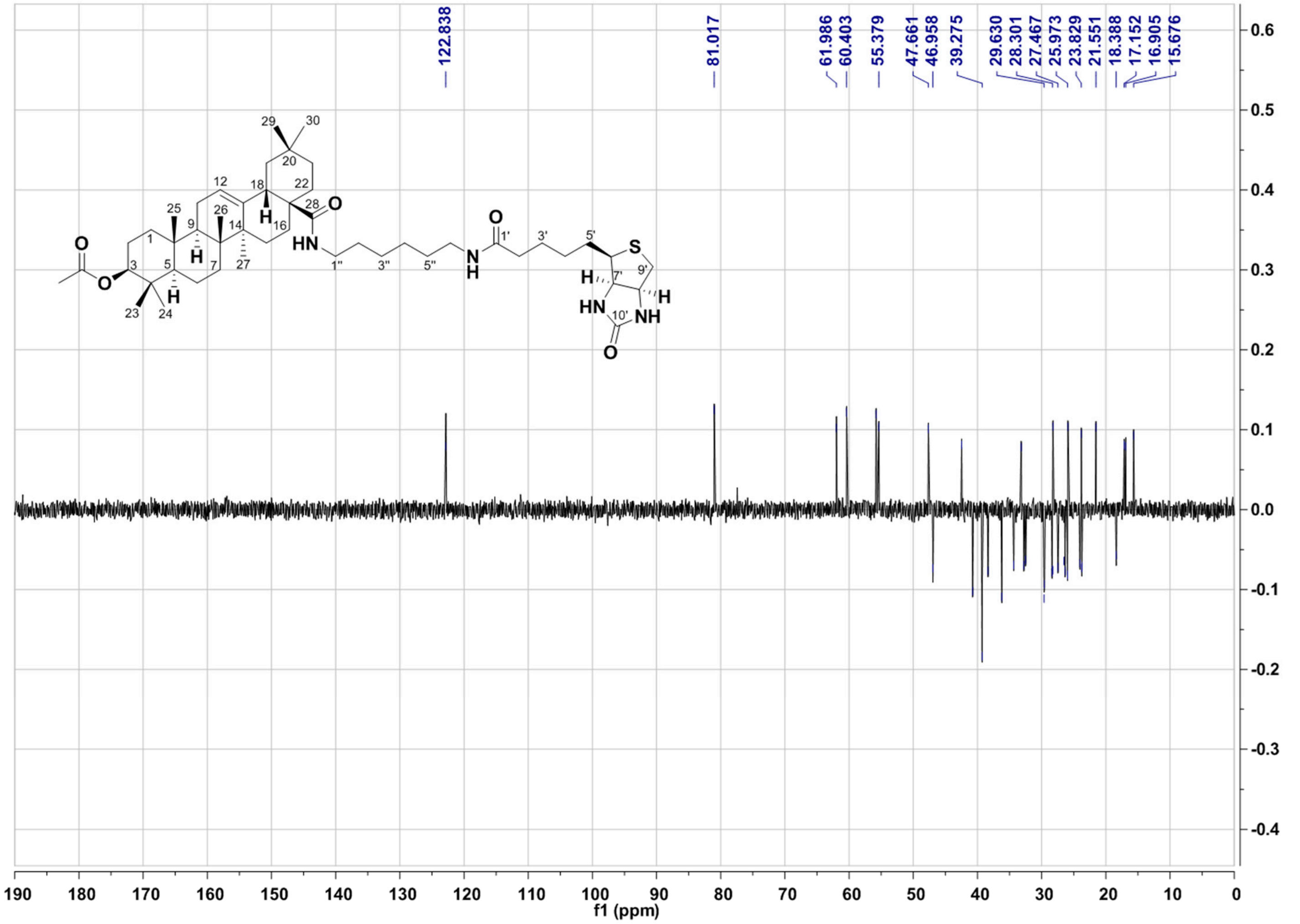

122.838
81.017
61.986
60.403
55.379
47.661
46.958
39.275
29.630
28.301
27.467
25.973
23.829
21.551
18.388
17.152
16.905
15.676
f1 (ppm)

Figure S6. DEPT-135 spectrum of Biotin-OAA ($CDCl_3$, 150 MHz)

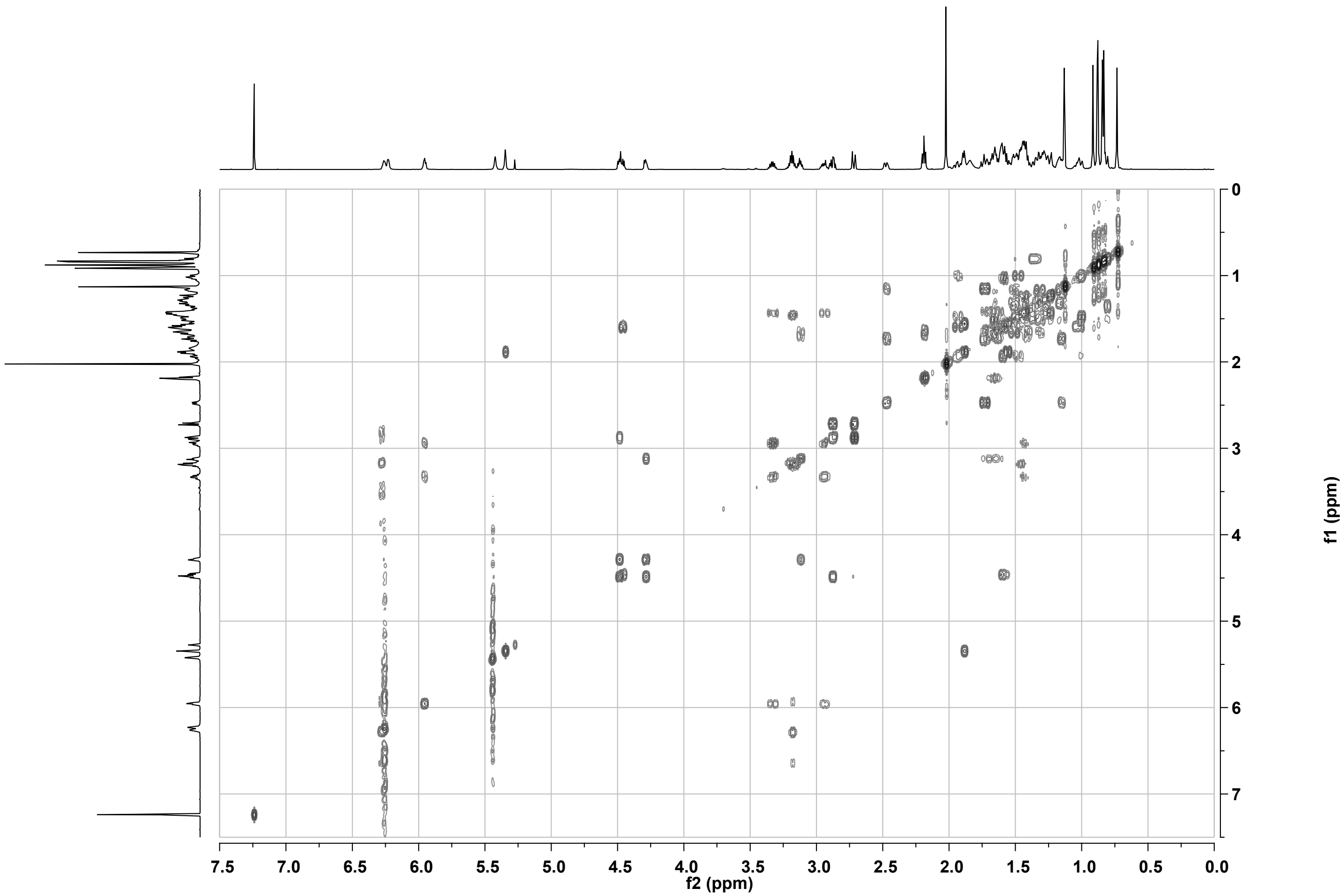

Figure S7. COSY spectrum of Biotin-OAA ($CDCl_3$, 600 MHz)

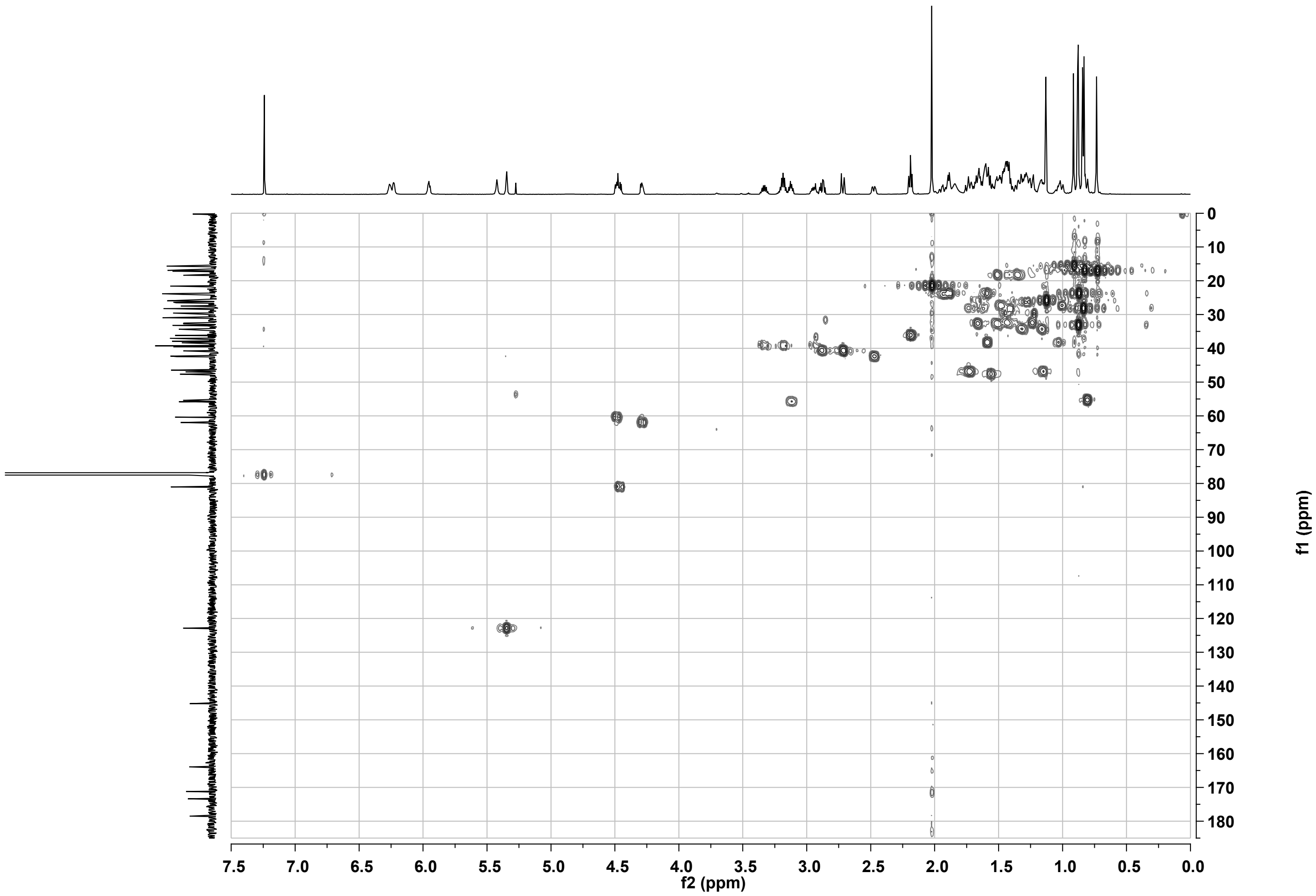

f2 (ppm)
f1 (ppm)

Figure S8. HMQC spectrum of Biotin-OAA ($CDCl_3$, 600 MHz)

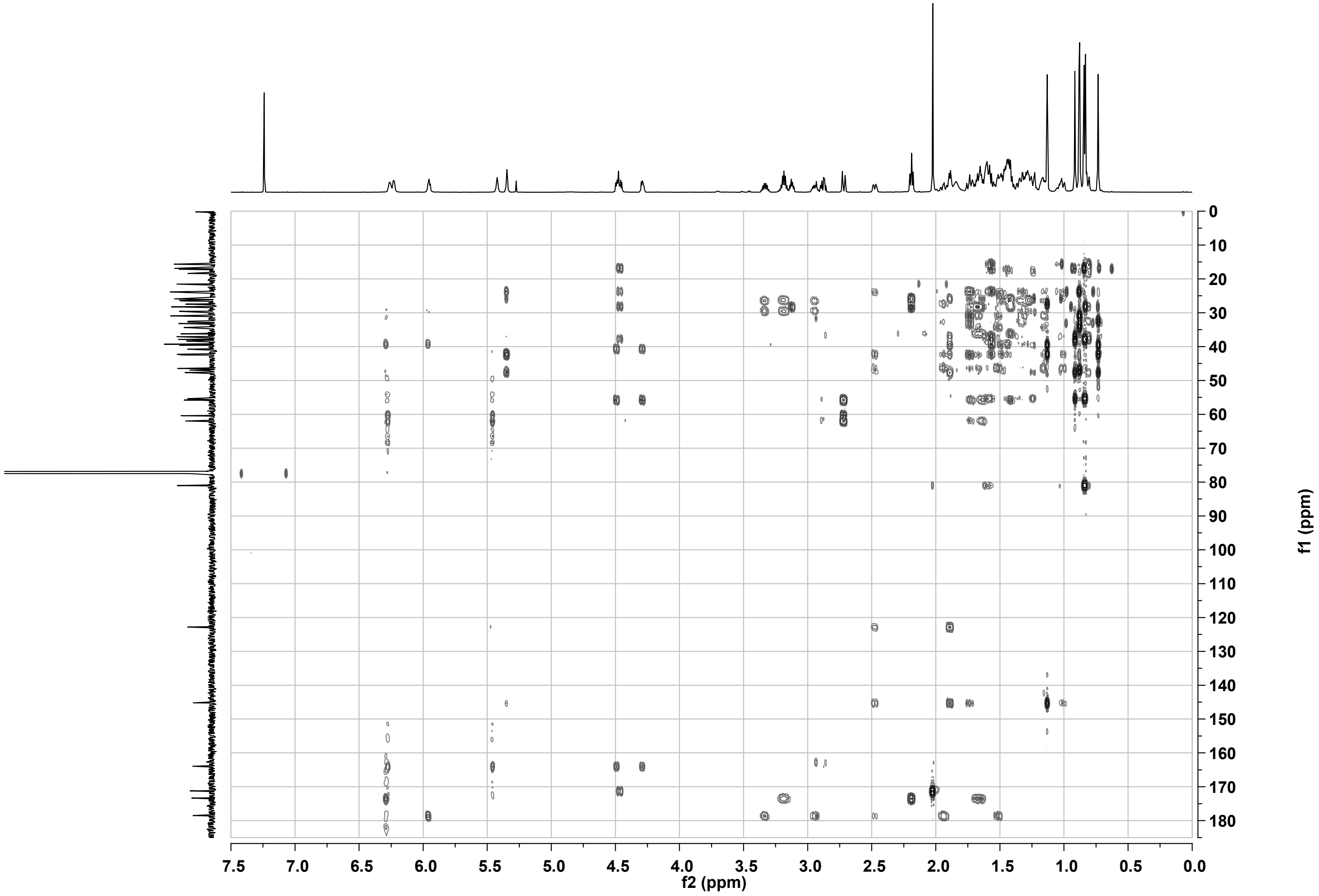
f2 (ppm)
f1 (ppm)

Figure S9. HMBC spectrum of Biotin-OAA ($CDCl_3$, 600 MHz)
